# A Systematic Review and Meta-Analysis of Impairment and Quality of Life in Children and Adolescents with Anxiety Disorders

**DOI:** 10.1007/s10567-024-00484-5

**Published:** 2024-05-23

**Authors:** Sophie J. Dickson, Ella L. Oar, Maria Kangas, Carly J. Johnco, Cassie H. Lavell, Ashleigh H. Seaton, Lauren F. McLellan, Viviana M. Wuthrich, Ronald M. Rapee

**Affiliations:** Macquarie University Lifespan Health and Wellbeing Research Centre, Sydney, 2109 Australia

**Keywords:** Impairment, Quality of Life, Anxiety, Children, Adolescents, Meta-analysis

## Abstract

Anxiety disorders are common, emerge during childhood, and pose a significant burden to society and individuals. Research evaluating the impact of anxiety on functional impairment and quality of life (QoL) is increasing; however, there is yet to be a systematic review and meta-analysis of these relationships in pediatric samples. This systematic review and meta-analysis were conducted to determine the extent of impairments in functioning and QoL that young people with anxiety disorders experience relative to their healthy peers, as well as sociodemographic and clinical moderators of these relationships. Studies were included when they compared young people (mean age range within studies 7–17 years) with a primary clinical anxiety disorder to a healthy comparison group and measured impairment and/or QoL via a validated instrument. A total of 12 studies met criteria for this review (*N* = 3,129 participants). A majority of studies (*K* = 9) assessed impairment as an outcome measure, and three assessed QoL outcomes. Meta-analysis of nine studies (*N* = 1,457 children) showed large relationships between clinical anxiety and life impairment (*g* = 3.23) with the strongest effects seen for clinician report (*g* = 5.00), followed by caregiver (*g* = 2.15) and child (*g* = 1.58) report. The small number of studies and diversity in methodology prevented quantitative investigation of moderating factors. In the systematic review of QoL outcomes, all three studies reported significantly poorer QoL for youth with anxiety disorders relative to unaffected peers. Findings support the importance of measuring functioning and QoL as outcomes in clinical research and practice among anxious young people.

This study is registered with PROSPERO under the identification number CRD42023439040.

Anxiety disorders are the most common mental health condition in children and adolescents. Prevalence estimates suggest that 6.5% of young people^1^ worldwide meet diagnostic criteria for an anxiety disorder in comparison with 2.6% for depressive disorders, 3.4% for attention deficit hyperactivity disorder, and 5.7% for disruptive disorders (Polanczyk et al., 2015). Pediatric anxiety disorders are associated with substantial economic burden, including both direct (e.g., treatment) and indirect (e.g., informal care) costs including adverse effects on the functioning of children (e.g., missed days of school), caregivers (e.g., time off work) and broader society (e.g., loss of productivity; Pollard et al., 2023). Indeed, the costs to society of pediatric clinical anxiety have been estimated to be 21 times greater than having no disorder (Bodden et al., 2008), with a recent meta-analysis suggesting that the total annual societal cost per anxious child is up to £4040 (2021 GPD) (Pollard et al., 2023). In addition to their high prevalence and cost, anxiety disorders are associated with substantial impairment in young people’s day-to-day functioning and a poorer quality of life.

## Impairment

Impairment refers to the degree to which a young person’s symptoms interfere with their ability to perform important aspects of their daily life (Rapee et al., 2012). Anxiety symptoms can result in difficulties across multiple areas of child functioning including their family, academic, and social life. For example, anxiety symptoms may impact young people’s relationships with caregivers and siblings, the completion of family routines (e.g., bedtime), and activities (e.g., parties, holidays, visiting friends or relatives) (Langley et al., 2014; Lyneham et al., 2013). In school settings, anxious young people show poorer academic performance, greater absenteeism, and are less likely to enter higher education following their secondary schooling than their non-anxious counterparts (de Lijster et al., 2018; Goodsell et al., 2017; Lawrence et al., 2015; Lee et al., 2009). Anxiety disorders are also negatively associated with peer relationships, with anxious young people reporting fewer friendships, greater loneliness and victimization, and poorer social competence (de Lijster et al., 2018). Impairment is a central feature of diagnostic criteria for most anxiety disorders and is one of the strongest drivers for seeking treatment (Becker et al., 2011). Changes in levels of impairment are also an indicator of treatment progress and success (see Dickson et al., 2022; Kreuze et al., 2018 for reviews). Despite the critical role impairment plays in the diagnosis and treatment of anxiety disorders, it is a surprisingly under researched area in children and adolescents (Langley et al., 2014).

In the extant literature, several measures of impairment have been developed for children and adolescents. These include both general functional impairment measures not specific to any disorder (e.g., Children's Global Assessment Scale (CGAS); Shaffer et al., 1983) and disorder-specific scales designed to assess the particular impact of anxiety disorders (e.g., Child Anxiety Impact Scale (CAIS) and Child Anxiety life interference Scale (CALIS; Langley et al., 2014; Lyneham et al., 2013). A number of widely used structured and semi-structured diagnostic interviews, such as the Anxiety Disorders Interview Schedule (ADIS; Albano & Silverman, 2016), also measure the impairment to inform diagnostic decision making. However, these clinician-rated interviews typically combine the ratings of symptom presence, severity, and impairment. While independent ratings of impairment can potentially be disentangled, this information is rarely reported (Rapee et al., 2012). Additionally, when impairment ratings from diagnostic interviews have been reported, they have shown relatively poor psychometric properties (Bird et al., 2000). A range of multi-informant (e.g., clinician, children, and caregiver) impairment measures have been developed, including the CGAS (Shaffer et al., 1983), which is clinician-administered, and the CALIS (Lyneham et al., 2013), the CAIS (Langley et al., 2014), and the Sheehan Disability Scale (SDS; Sheehan et al., 1996) which offer both caregiver and child self-report versions. Consistent with informant discrepancies reported in the broader child anxiety literature, relatively modest agreement has been observed between young person and caregiver ratings of impairment, with clinician ratings often aligning most closely with caregivers (De Los Reyes et al., 2011; Dickson et al., 2022; Lyneham et al., 2013).

Mixed findings have been documented between studies that have examined the correlation between impairment and demographic and psychological variables, including age, gender, ethnicity, socioeconomic status, type of anxiety diagnosis, and medication status. While some pediatric studies have found greater overall impairment and interference in academic and social functioning among older children (Langley et al., 2014; Whiteside, 2009), other studies have found no age differences (Langley et al., 2014; Lyneham et al., 2013). Mixed gender effects have also been observed. For example, Lyneham et al. (2013) found that female young people with anxiety reported greater impairment than their male counterparts. However, other studies have not found gender differences in impairment (Langley et al., 2004, 2014; Whiteside, 2009). Mixed effects have also been documented for ethnicity. While some studies have not found differences in the levels of impairment based on ethnicity (Langley et al., 2004), others (e.g., Langley et al., 2014) have found that Hispanic children and adolescents with anxiety experienced greater overall and social impairment than other ethnic groups.

## Quality of Life

Quality of life (QoL) is a multidimensional construct reflecting the positivity with which a young person views his/her life circumstances and state (Olatunji et al., 2007). It extends beyond anxiety symptoms to include an overall sense of well-being and life satisfaction and encompasses physical, psychological, and social functioning. While it is commonly used interchangeably with the term impairment, these two constructs are conceptually distinct (Rapee et al., 2012). A substantive body of research has investigated the relationship between anxiety and QoL in adults; however, to date, QoL in anxious young people has been largely neglected. The first and only meta-analysis to compare QoL in adults between anxious and non-clinical controls identified 23 studies (*N* = 2892) and, as expected, yielded large effect sizes suggesting poorer QoL among anxious adults (Olatunji et al., 2007). Not surprisingly, among the few studies conducted in young people, overall QoL has also been found to be inversely associated with anxiety in young people (Öztürk et al., 2018; Raknes et al., 2017). In cross-sectional studies, and in comparison with non-anxious children and adolescents, anxious young people have been found to report poorer QoL across multiple dimensions (e.g., physical well-being, psychological well-being, autonomy and parent child relations, social support and peers and school environment (Raknes et al., 2017; Telman et al., 2017). A systematic review and/or meta-analysis that synthesizes these disparate findings is needed.

Several child and caregiver QoL measures are available, which assess both global QoL and specific dimensions. These measures are designed to be used across a broad range of physical (e.g., cancer, diabetes, kidney disease) and mental health problems (e.g., Autism) such as the Pediatric Quality of Life Scale (PedsQL; Varni et al., 2001) and the Pediatric Quality of Life Enjoyment and Satisfaction Questionnaire (PQ-LES-Q; Endicott et al., 2006). The three dimensions, physical, psychological, and social functioning are commonly included in the majority of QOL measures in this field. Reflecting the healthcare shift toward considering young people’s priorities and preferences alongside their symptoms, recent mental health-specific QoL measures like the Self-Report Quality of Life—Child and Youth Mental Health Instrument (QoL-ChYMH; Celebre et al., 2021; Stewart et al., 2020) have been developed.

Only a small number of studies have explored the factors associated with QoL in anxious young people with mixed effects reported. One such study, conducted by Ramsawh and Chavira (2016), found that greater comorbidity, anxiety severity, and specific type of anxiety symptomatology (e.g., physical and social anxiety symptoms) were correlated with poorer QoL, while age, gender, and ethnicity were not significantly associated with QoL. In contrast, Raknes et al. (2017) found that older age, female sex, lower socioeconomic status, and negative life events were associated with poorer QoL. Given the growing volume of studies assessing QoL, and sociodemographic moderators of QoL, in anxious youth, a comprehensive review of this literature provides an opportunity to clarify these relationships.

While individual studies have documented that anxious young people experience significant impairment in their functioning at home with their family, at school, and with peers (de Lijster et al., 2018; Goodsell et al., 2017; Langley et al., 2014; Lawrence et al., 2015), no comprehensive synthesis has been conducted to aggregate these findings and quantify the overall extent of impairment associated with anxiety disorders in young people. Similarly, the degree to which anxiety disorders affect the QoL of young people in comparison with healthy peers remains unclear. A systematic review and quantitative analysis of these relationships is important to understand the impact of anxiety disorders on child and adolescent functioning and overall QoL. In addition, this synthesis of the literature may allow a more comprehensive analysis of potential sociodemographic moderators of impairment and QoL, including age, gender, reporter (e.g., child, caregiver or clinician), anxiety disorder subtype, comorbidity, and type of assessment measure used (e.g., global versus anxiety disorder-specific). Hence, the primary objective of this study was to conduct a systematic review and meta-analysis of impairment and QoL in young people with clinical anxiety disorders compared to unaffected healthy control groups.

## Method

The protocol for this study was registered and is accessible at PROSPERO 2023 CRD42023439040. This systematic review and meta-analysis followed the Preferred Reporting Items for Systematic Reviews (PRISMA) guidelines, as detailed by Page et al., (2021).

### Eligibility Criteria

Studies were eligible for this review if they were peer reviewed, published in English, and included a sample of children and/or adolescents with a mean age between 7 and 17 years, regardless of whether some participants fell outside of this specific range. Studies were required to include children and/or adolescents diagnosed with a primary anxiety disorder as defined by any version of the Diagnostic and Statistical Manual of Mental Disorders (American Psychiatric Association, 1980, 1987, 1994, 2000, 2013) or the International Classification of Diseases (World Health Organization, 1993, 201,9). Disorders categorized as anxiety disorders have changed across versions of the DSM and ICD. In our review, anxiety disorders included generalized anxiety disorder, social anxiety disorder, specific phobia, separation anxiety disorder, panic disorder, agoraphobia, or selective mutism, as determined by a diagnostic interview or based on participants exceeding a clinical cut-off score on a validated measure. In previous versions of the DSM, both posttraumatic stress disorder (PTSD) and obsessive compulsive disorder (OCD) were classified as anxiety disorders. In our study, we excluded studies that specifically focused on OCD or PTSD due to our assumption that these disorders would be associated with greater levels of impairment and lower QoL compared to DSM-5 anxiety disorders, thus potentially leading to an overestimation of the overall impact of anxiety disorders. However, we included studies if these disorders were part of a broader sample recruited for anxiety disorders, reflecting their classification at the time. Studies focused on participants with subclinical symptoms were excluded unless separate outcome data were available for clinical and subthreshold samples. We included studies where participants had other comorbid diagnoses (e.g., Obsessive–Compulsive Disorder, Illness Anxiety Disorder, Major Depressive Disorder), provided the sample had a primary diagnosis of anxiety. We excluded studies that focused on samples where every participant had a specific homogenous co-occurring psychiatric or physical health diagnosis in addition to anxiety (e.g., autism, intellectual/learning disabilities, asthma). However, if a study did not deliberately select for co-occurring diagnoses but included participants who happened to have them (as long as the primary focus was on anxiety and not all participants had the co-occurring condition), these studies were considered eligible. We considered studies across all design types (e.g., longitudinal, psychometric evaluation, national survey data), as long as they compared participants with clinical anxiety to a healthy comparison group. Healthy control samples could be established either by confirming the absence of any psychiatric or physical diagnosis in the group or by showing no indicators of these diagnoses based on the study’s screening methods. The outcomes of interest were impairment and QoL. To be included in the meta-analyses, studies had to report the means and standard deviations (SDs) for one or more of these outcomes or provide data that would allow us to compute the relevant statistics.

#### Impairment

For the purposes of this review, eligible measures of impairment included scales that had been validated in at least one study assessing their psychometric properties and could generate a single summary score for impairment across multiple domains of life (e.g., at home, at school, with peers). Measures could be those specifically designed and validated for use with anxious children and adolescents, or general measures validated for children and adolescents with a broad range of physical and/or mental health problems. If a measure’s protocol allowed for subscale scores to be summed into a total score, the total score was included in our analysis. Our decision to include only global measures of impairment was driven by the relatively nascent and diverse state of domain-specific measures of impairment (e.g., impairment in family functioning can be characterized by measures of family conflict, caregiver burden, sibling relationship quality). These measures often lack standardization and validation and cover heterogeneous domains and issues (Dickson et al., 2022; Etkin et al., 2023a), making comparisons across studies and combining effect sizes across these diverse domains problematic for meta-analysis.

Notably, a large number of studies in the anxiety field use the Anxiety Disorder Interview Schedule for Children (ADIS; Albano & Silverman, 1996) to diagnose child/adolescent anxiety. This diagnostic interview generates a clinician severity rating (CSR) which combines symptom severity with impairment. Hence, because of this confound, we excluded CSR as an indicator of impairment for the purposes of this review.

#### Quality of Life

Eligible measures of QoL were those that had been validated and generate either a single summary score for QoL across various domains, subscale scores for specific dimensions of QoL (e.g., physical or emotional), or both. In contrast to life impairment, we considered multidimensional measures of QoL due to their long-standing standardization and validation in this field.

### Search Strategy

Relevant studies were identified through database searches on PsycINFO, Medline, PubMed, and Web of Science (July, 2023). The search consisted of a variety of key terms and their plural forms (“adolescent/adolescence”), alternative spellings (“generalized/generalized anxiety”), and synonyms used interchangeably in the literature (“life impairment/functioning”). Additionally, the search incorporated commonly used measures such as the ‘Children’s Global Assessment Scale’ and ‘PedsQL,’ not as an exhaustive list but to bolster the retrieval of relevant studies across diverse research contexts. When the database allowed selection filters, our search was limited to peer-reviewed studies in English, per our eligibility criteria. Search strategies can be found in Appendix A.

### Selection Process

Studies identified through the search had duplicates removed initially in Endnote, then were imported into Covidence (Covidence, 2017), where remaining duplicates were automatically removed. All subsequent screening procedures were conducted within Covidence. Two reviewers (CL and AS) independently screened the titles and abstracts of studies against the inclusion criteria. Studies where both reviewers agreed on inclusion or where initial disagreements were resolved in favor of inclusion were proceeded to full-text review. Downloaded full texts were screened independently by the same two reviewers, who recorded reasons for exclusion. Any disagreements during either stage were resolved first through discussion, and if agreement could not be reached, a third reviewer was consulted to make the final decision (SD). If multiple reports used the same sample, we included only the most recently published one so that our review concentrated on unique studies rather than multiple reports of the same data.

Recognizing that impairment is often considered a secondary outcome in studies and therefore might not be mentioned in abstracts, we anticipated a challenge in identifying eligible studies through traditional abstract review. To address this, we adapted our screening procedure based on advice from a previous meta-analysis on impairment which successfully identified additional papers using the same approach (Dickson et al., 2022). We assigned a unique identifier to papers that appeared to meet all the inclusion criteria except for explicit mention of an impairment outcome in their abstracts. After the abstract review, we examined the methods sections of these flagged papers to check for the inclusion of a global measure of life impairment.

### Data Extraction

For data extraction, two reviewers (CL and AS) independently extracted variables relating to:

#### General Study Information

Year, country of origin, aims, design, sample size.

#### Participant Characteristics

Mean age (range), %female, study-entry primary anxiety diagnosis (specific or any), diagnostic method for anxiety, comorbidity. Study-entry anxiety diagnosis referred to the specific anxiety disorder for which participants were recruited. When studies recruited for a single anxiety disorder, such as social anxiety disorder, we noted the specific diagnosis. Studies without a specific anxiety disorder type were categorized as ‘Any.’ Comorbidities were identified, including any non-entry criteria diagnoses.

#### Comparison Group

Type, validation details.

#### Outcome

Type (life impairment or QoL), measurement scale, direction of effect (higher score represents better or worse outcome), reporter (caregiver, child, clinician), timepoint (if applicable), mean and standard deviation of outcome scores for both clinical anxiety and healthy control group. Our protocol for handling multiple measures of the same outcome within the same study was to prioritize the most commonly used measure across the analysis. However, we found that each study in our review only provided data for a single eligible measure. For clinician-administered measures of impairment that offered both caregiver and child ratings, we prioritized the caregiver rating if a combined score was not available. Two studies included both caregiver and child versions of the same scale (Lyneham et al., 2013; Whiteside, 2009). For these studies, we prioritized the caregiver rating because they typically align more closely with clinician reports compared to child reports (De Los Reyes et al., 2011). Since clinician reports constituted the majority of our data, this approach was taken to maximize consistency. A sensitivity analysis substituting caregiver data with child data yielded a slightly smaller effect, although it remained large, highly significant, and considerably heterogeneous (see Appendix B). This indicates that prioritizing the caregiver data did not significantly impact the overall findings.

### Quality Coding

Two reviewers (CL and AS) independently evaluated each study’s quality using the Checklist for Assessing the Quality of Quantitative Studies (Kmet et al., 2004). The checklist comprises 14 targeted questions (for example, “were subject and comparison group/s sufficiently described?”) covering key quality areas: study design, methodology, sample size, analyses, completeness of results reporting, and whether the conclusions were supported by the results. For each question, reviewers responded by assigning a score of “yes” (2 points), “partial” (1 point), or “no” (0 points). A total quality score was obtained by summing the scores of individual items and dividing by the maximum possible score, yielding a quality score from 0 to 1. Checklist items that were irrelevant due to the study design (e.g., random allocation) were marked as n/a, leaving the total score unaffected. In line with previous meta-analyses, we used a score of 0.70 or higher as indictor of adequate study quality (Christina et al., 2021). Studies were not excluded based on quality ratings and, however, were utilized when prioritizing and interpreting the results.

### Planned Analysis

Meta-analysis was performed with Review Manager version 5.4 (Cochrane Collaboration, 2020). To account for expected variations across trials due to study characteristics, we employed the DerSimonian and Laird inverse-variance method with a random-effects model. We calculated Hedge’s g statistic as the between-group effect size and interpreted it according to guidelines (Cohen, 1998), with *g* = 0.2 as a small, *g* = 0.5 as a medium, and *g* = 0.8 as a large effect size. In this study, higher scores indicate greater levels of impairment. For scales where higher scores represented better functioning, we reversed the direction of the effect by subtracting the mean from the maximum possible score for that scale prior to standardization. The I^2^ statistic was used to test percentage heterogeneity of the effect sizes. We interpreted I^2^ level according to Cochrane’s guidelines: 0%–40% (might not be important), 30%–60% (moderate), 50%–90% (substantial), and 75%–100% (considerable). When necessary data were unavailable in the published paper, we first tried to contact the authors of the original papers for missing data or when we required a specific subset of the data reported (e.g., separating primary anxiety from mixed samples). When the outcome data appeared only in graphical form, we used WebPlotDigitaliser software for extraction (Rohatgi, 2022). We imputed missing SDs for three studies (Alfano, 2012; Benjamin et al., 1990; Sheehan et al., 2010) using the average SD from studies in our meta-analysis that had complete data for the identical measure (Higgins et al., 2023). One study (Alfano, 2012) had multiple eligible experimental groups. To best represent the effect of anxiety on the outcome, we selected the “pure” GAD group for analysis, excluding a GAD group with additional comorbidities (other anxiety disorders and non-anxiety disorders), even though this group would have been eligible if it were the only comparison available. Our protocol initially outlined the use of funnel plots and Egger’s regression to assess for publication bias. However, adhering to advice from Page et al. (2023), we chose not to employ these tests because we had fewer than 10 studies, which would compromise the power to detect true asymmetry.

## Results

**Fig. 1:**
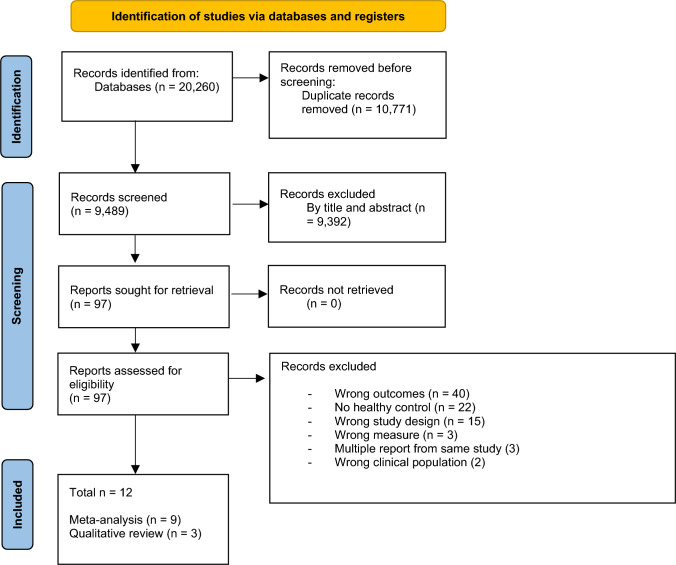
PRISMA flow diagram of literature search and study selection. Note. From Page et al. (2021)

The method for identifying, screening, and including studies is depicted in Fig. 1. From the initial search, we identified 9,489 studies after the removal of duplicates. There was moderate inter-rater agreement for the title/abstract review (*k* = 0.45) and substantial agreement during the full-text review (*k* = 0.78). Out of the 97 full-text articles assessed, 85 did not meet the criteria. The most common reason for exclusion was that they did not measure a relevant outcome (*n* = 40). Within this subset, a common problem was that although studies may have included relevant measures (e.g., CGAS), separate results were not reported for the healthy control group (*n* = 12). Upon contacting the authors, we learned that in many cases, this measure had not been administered to the healthy control sample, often due to the assumption of no impairment in this group. Given our focus on controlled comparisons, the absence of this data resulted in the exclusion of these studies.

### Study Characteristics

Twelve studies, comprising 3,129 participants (1,072 clinical, 2,057 controls), met all criteria and underwent quality evaluation: nine for meta-analysis (impairment) and three for systematic review (QoL). Table 1 includes the characteristics of the included studies. There was almost perfect inter-rater agreement on extracted outcome data (*k* = 0.90).

**Table 1:** Characteristics of included studies

		Country	*N*	Age mean (range)	Study-entry Anxiety Dx	Comorb	Healthy Control	Outcome	Scale	Reporter	Quality Rating
1.	Alfano, 2012	USA	37	9 (6 to 11)	GAD	none	Community	Impair	^a^CASAFS – School CASAFS – Peers CASAFS – Family CASAFS – Home	Child	1.00
2.	Beidel et al., 2007	USA	106	14 (13 to 16)	SocAD	OAD, NAD	Opposite trait	Impair	K-GAS	Clinician	0.96
3.	Benjamin et al., 1990	USA	297	9 (7 to 11)	Any	OAD, NAD	Community	Impair	CGAS	Clinician	0.73
4.	Hansen et al., 2014	NOR	74	11 (7 to 13)	Any	OAD, NAD	Community	Impair	CGAS	Clinician	0.95
5.	Lyneham et al., 2013	AUS	80	10 (6 to 17)	Any	OAD, NAD	Opposite trait	Impair	CALIS-P CALIS-C	Caregiver Child	1.00
6.	Manassis et al., 2009	CAN	130	10 (8 to 12)	Any	OAD, NAD	Community	Impair	CGAS	Clinician	0.88
7.	Öztürk et al., 2018	TUR	97	10 (8 to 12)	Any	OAD	Community	QoL	PedQL-P PedQL-C	Caregiver Child	0.95
8.	Raknes et al., 2017	NOR	1,470	NR (12 to 17)	Any	NR	Community	QoL	KINDL-R – Phys KINDL-R -Emo KINDL-R – Self KINDL-R – Fam KINDL-R – Friend KINDL-R -School	Child	0.86
9.	Schniering et al., 2023	AUS/ USA	276	15 (11 to 18)	Any	OAD, NAD	Opposite trait	Impair	ALIS-I	Child	1.00
10.	Sheehan et al., 2010	USA	128	13 (6 to 17)	Any	OAD	Community	Impair	SDS-C	Child	0.77
11.	Telman et al., 2017	NLD	105	12 (NR)	Any	OAD	Community	QoL	KIDSCREEN-27 – PW KIDSCREEN-27 PsyW KIDSCREEN-27 – APCR KIDSCREEN-27 – SSP KIDSCREEN-27—SE	Caregiver	1.00
12.	Whiteside, 2009	USA	97	12 (5 to 19)	Any	OAD, NAD	Community	Impair	CSDS-P CSDS-C	Caregiver Child	0.82

*Note.* ^a^CASAFS subscales sum to a global score as per protocol

Abbreviations: *USA* United States of America; *NOR* Norway; *AUS* Australia; *CAN* Canada; *TUR* Turkey; *NLD* The Netherlands; *GAD* generalized anxiety disorder; *SocAD* social anxiety disorder; *OAD* Other anxiety diagnoses; *NAD* non-anxiety diagnoses; *ALIS* Adolescent Life Interference Scale; *C/K-GAS* Children’s Global Assessment Scale; *CALIS-P/C* The Child Anxiety Life Inference Scale – parent and child version; *PedsQL-C/P* Pediatric Quality of Life Inventory; *KINDL-R – Phys/Emot/Self/Fam/Friends/School* Questionnaire for Measuring Health-Related Quality of Life in Children and Adolescents Revised Version – Physical Health/Emotional Health/Self-esteem Health, Family Health/Friends Health/School Health); *SDS* Sheehan Disability Scale – parent and child version; *CSDS-C/P* Child Sheehan Disability Scale – parent and child version; *KIDSCREEN-27 – PW/Psy/APCR/SSP/SE* KIDSCREEN-27 Physical wellbeing/Psychological wellbeing/Autonomy and Parent–Child Relations/Social Support and Peers/School Environment

#### Impairment

Nine studies comprised 1,457 participants (852 clinical, 605 controls). Six studies recruited healthy controls from the broader community, while three studies specifically advertised for community controls displaying traits considered the opposite from anxiety, such as “confident children.” Participants had an average age of 11 years (SD = 2.3, range from 5 to 19 years) and 44.5% were female. Impairment was predominantly assessed via clinician reports. Of the nine impairment measures, seven studies used general functional impairment measures (e.g., CGAS), while two used measures that assessed impairment specifically related to anxiety (e.g., CALIS). Comorbidities were common, with eight studies reporting multiple anxiety disorders, and seven reporting additional non-anxiety disorders. For the meta-analysis, 9 studies generated large effect sizes ranging from *g* = 0.9 to 6.3.

#### QoL

Three studies comprised 1,672 participants (220 clinical, 1,452 controls). All three studies recruited healthy controls from the broader community. Participants had an average age of 11 years (SD = 1.6, range 8 to 17 years) and 53.9% were female. All of the QoL scales measured specific dimensions of QoL (e.g., physical, emotional, social, school). In two of the studies, participants had multiple anxiety diagnoses, while the remaining study did not report comorbidities.

### Study Quality

Quality evaluations can be found in Table 1. The overall quality of the included studies ranged from 0.73 to 1.0 with a mean value of 0.91 (SD = 0.09) on a scale from 0 to 1. All twelve studies surpassed the benchmark for adequate quality (scores ≥ 0.70).

### Meta-analysis of Life Impairment in Children and Adolescents with Anxiety Compared to Healthy Controls

**Fig. 2:**
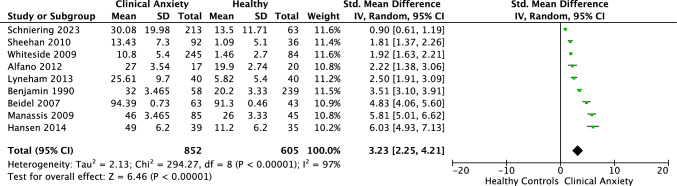
Forest plot: life impairment in clinical anxiety versus healthy controls

**Fig. 3:**
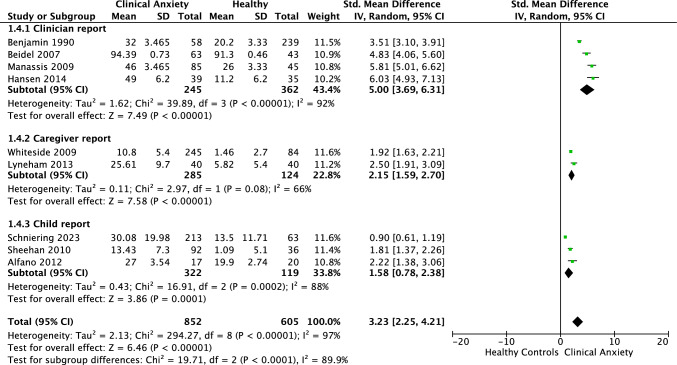
Forest plot: life impairment in clinical anxiety versus healthy controls by reporter type

Meta-analysis of nine studies, comprising 1,457 participants, revealed a significant large effect difference between clinical anxiety and controls on life impairment of *g* = 3.23 (95% CI = 2.25, 4.21; *Z* = 6.46 *p* < 0.001). Heterogeneity was considerable, as indicated by *I*^2^ value of 97% (See Fig. 2). As such, we conducted exploratory analyses of differences in results separated by reporter type. Per Cochrane guidelines, at least ten studies are advised when undertaking subgroup analysis (Deeks et al., 2023). Considering our distribution with 4 clinician reports, 3 child reports, and 2 caregiver reports, this separation wasn’t powered for definitive conclusions. However, the marked differences in effect sizes across the different reporters warranted mention. Statistical tests confirmed a difference in outcomes by reporter type (*p* < 0.001). Each group exhibited a large, significant effect size, with clinicians reporting the largest effect (*g* = 5.00), followed by caregivers (*g* = 2.15) and children (*g* = 1.58). While data separation reduced heterogeneity, substantial unexplained heterogeneity remained between the studies within each group (clinician *I*^2^ = 92%; caregiver *I*^2^ = 66%; child *I*^2^ = 88%). Interested readers can view this analysis in Fig. 3, but we advise interpreting the findings with caution.

### Systematic Review of Quality of Life in Children and Adolescents with Anxiety Compared to Healthy Controls

Given that only three studies assessed QoL, we felt that quantitative combination of their results would be potentially misleading and therefore a qualitative synthesis is presented here. Based on qualitative evaluations, three studies reported significantly lower levels of QoL across multiple dimensions in anxious children and adolescents relative to their healthy counterparts. Specifically, Öztürk et al. (2018), Raknes et al. (2017), and Telman et al. (2017) using the PedsQL (Varni et al., 2001), the KINDL (Ravens-Sieberer & Bullinger, 1998), and the KIDSCREEN-27 (The KIDSCREEN Group, 2006), respectively, showed larger differences on the dimensions of emotional health (*d* = 0.93 to 2.2) and physical health (*d* = 0.68 to 1.87) than the dimensions of social health (*d* = 0.53 to 1.29) or school (*d* = 0.46 to 0.62). The study of Öztürk et al. (2018) was the only study that reported QoL separately from pediatric and caregiver perspectives, showing similar effects across dimensions, yet with consistently slightly larger effects reported on the child form: for physical health (child* d* = 1.87 vs. caregiver *d* = 0.90), emotional health (child *d* = 2.15 vs caregiver *d* = 1.97), social health (child *d* = 0.77 vs caregiver *d* = 0.72), school (child *d* = 0.62 vs. caregiver *d* = 0.56), and total scale score (child *d* = 1.59 vs parent *d* = 1.38).

## Discussion

Over the past decade, the association between anxiety disorders and poor functioning and overall QoL among pediatric samples has received increasing attention. However, given the dearth of quantitative reviews in this field, we conducted the first systematic and meta-analytic review to synthesize the findings from this growing body of literature, and to determine the extent of impairment in functioning and QoL that young people with anxiety disorders experience relative to their healthy peers. A total of 12 studies met criteria for this review. A majority of studies (*K* = 9) focused on impairment as the outcome measure in which the results were available for both anxious and non-anxious pediatric samples (i.e., studies which included both anxious and control/comparison conditions), while only three studies were identified which met review criteria and assessed QoL.

Children and adolescents with anxiety disorders reported significantly greater impairment in functioning relative to non-anxious youth, with an overall large effect size (*g* = 3.23), and with considerable heterogeneity between studies. Preliminary findings further revealed that although calculated effect sizes were large across each type of reporter, clinician reports evidenced the strongest effects (*g* = 5.00) followed by caregiver (*g* = 2.15) and child (*g* = 1.58) reports. This outcome is compatible with the findings from the broader pediatric anxiety literature which has shown that clinician and caregiver reports tend to show larger discrimination between samples or across treatment relative to child reports (Reardon et al., 2018; Spence, 2018). However, as noted, the effect sizes even for self-reports from the young person remained large in the current review, demonstrating that even anxious young people, themselves, perceive high levels of life impairment. Although the comparison between raters needs to be interpreted cautiously due to the small number of studies, it is interesting to note that the group differences reported by clinicians appear to be considerably larger than other reporters. Assuming that clinicians are often not blind to group membership, it is possible that they are affected by an a-priori assumption that anxious young people are heavily impacted by their anxiety. Overall, the quality of studies was found to be very good which provides further confidence in the current findings. These findings attest to the importance of targeting improvements in functioning in addition to a reduction in symptom severity in treatment interventions for children and adolescents with anxiety disorders (Dickson et al., 2022; Rapee et al., 2012; Wu et al., 2016).

Given the large heterogeneity identified across studies, it is likely that these effects are underpinned by several moderators. Unfortunately, the small number of quantitative studies conducted to date meant that we were unable to evaluate the impacts of potential moderators. It is noteworthy that a majority of the studies (*K* = 7 of the 9 studies) which reported impairment outcomes included samples with a range of anxiety disorders, while one study was based on a sample diagnosed specifically with generalized anxiety disorder (GAD; Alfano, 2012), and another study was based on a pediatric sample with social anxiety (Beidel et al., 2007). Collectively, these findings indicate that child and adolescent samples experiencing a range of different anxiety disorders likely report significant impairments in functioning relative to non-anxious pediatric samples. However, at this stage, differences in impairment between specific anxiety disorders remain unclear. Impairment in functioning is probably common across all pediatric anxiety disorders, although this conclusion needs to await a larger research base.

Given the increasing importance of factors such as peer relationships, self-concept, and test results as young people move into later adolescence (Rapee et al., 2019), it might be predicted that the impact of anxiety disorder would increase with age. Similarly, the fact that a greater proportion of boys than girls with anxiety disorders seem to be brought for treatment might indicate a larger perceived impairment associated with anxiety among males (Rapee et al., 2023). Perhaps surprisingly at least some individual studies have failed to show differences in the impact of anxiety based on age (Langley et al., 2014; Lyneham et al., 2013) or sex (e.g., Langley et al., 2014; Whiteside, 2009). Unfortunately, the number of studies in our review was insufficient to be able to analyze the influence of these moderators. Further, age ranges in most studies are relatively limited and few studies break down their means by age or sex. Considerably more research evaluating the relationship between pediatric anxiety disorders and life impairment is needed to address the influence of demographic moderators and this question may also be better addressed using different analyses such as individual participant data meta-analysis.

The current findings should also be interpreted in relation to the measures used to assess impairment. Most research which has assessed impairment in children and adolescents relied on administering general (i.e., not disorder-specific) and global measures such as the CGAS. Use of consistent measures across a field is valuable to allow between study and potentially between disorder comparisons. However, this consistency comes at the cost of specificity. Hence, critical information may be missed that could enhance anxiety treatment planning and evaluation of progress (Etkin et al., 2023b). Broad overarching measures of functioning (such as CGAS) also risk missing the nuances of measures that break down functioning into specific domains. For example, anxiety may facilitate greater functional impairment within a school setting relative to home, whereas a disorder such as depression might involve more consistent impact across domains. Such profile differences might even extend to the level of specific types of anxiety disorders. However, this has yet to be empirically evaluated within the pediatric field. Future research should aim to implement consistent measures across studies that, nonetheless, encompass detailed assessment of functional impairment across a range of separate domains.

Interestingly, in the current review, no study was identified that measured both impairment in functioning and QoL. This may further reflect the nuanced approach researchers have adopted in this field. That is, whereas some scholars may be using more global scales of impairment, others may be relaying on multidimensional QoL scales to index functioning across specific domains (e.g., Öztürk et al., 2018). To this end, in the current review, only three studies compared children and/or adolescents with anxiety disorders to healthy controls on QoL (Öztürk et al., 2018; Raknes et al., 2017; Telman et al., 2017). The three studies evaluated QoL using different scales but converging on key dimensions including physical, emotional, social, and school. Across these dimensions, each study reported moderate to large effect size differences, indicative of poorer QoL in the group with anxiety (*d* = 0.46 to 2.2). The single study offering both child self- and caregiver reports on QoL revealed a potential discrepancy, with children reporting somewhat larger effects than caregivers across every dimension and in the overall QoL (child *d* = 1.59 vs parent *d* = 1.38) (Öztürk et al., 2018). Although only one study, it is interesting to note the difference of this pattern from reports on functional impairment, where caregivers show stronger discrimination between anxious and non-anxious groups. If this pattern was demonstrated in other studies, it might underscore an important difference in the two constructs and the role of caregivers in assessing them. While impairment involves disruptions in daily functioning that are often observable, allowing caregivers to serve as direct raters, QoL is definitionally internal and subjective (Olatunji et al., 2007). When caregivers assess their child’s QoL, they do so only as ‘proxy raters’ (e.g., Telman et al., 2017), thereby potentially relying more directly on their child’s descriptions (Öztürk et al., 2018). On the other hand, functional impairment is generally assessed as a negative characteristic and may therefore maximize the tendency to “fake good” (De Los Reyes et al., 2015; Kendall & Chansky, 1991; Schniering & Lyneham, 2007) from anxious young people.

This is the first review to synthesize the findings from studies that included outcome data on both functional impairment and QoL from young people with anxiety disorders and healthy control group comparisons. The quality of the included studies was strong, thus supporting confidence in the findings. The explicit focus on anxiety disorders in line with current diagnostic frameworks allows meaningful clinical conclusions to be drawn, but does mean that related conditions including PTSD and OCD had to be excluded.

We acknowledge several limitations associated with this review. The relatively small number of studies which include healthy comparison conditions in the anxiety pediatric literature attests to the shortcomings of this field. Almost twice as many studies (*K* = 22) were excluded than included at the full-text phase of this review because no healthy control group data were included. There is a current inherent assumption that non-anxious (healthy) young people have uniformly better levels of functioning across multiple domains. Yet as aforementioned, there is a paucity of studies that have empirically compared anxious against non-anxious young people across multiple domains of functioning. There is a potential confound in the recruitment of healthy control groups in three studies, which specifically sought ‘confident’ or ‘friendly’ young people. Such criteria may not represent the normative range of the broader non-clinically anxious population, particularly in early teenage years, where self-conscious feelings and emotions are heightened (Rapee et al., 2023; Westenberg et al., 2007). The review was restricted to papers published in the English language in peer review journals. Hence, it is possible we may have missed some existing research. Finally, studies that included dimensional assessment of the relationship between anxiety severity and impairment and/or QoL within anxious samples were excluded due to the different conceptual focus of this research. Again, this limited the number of suitable studies.

Notwithstanding these limitations, the findings from this review accentuate the negative relationship between anxiety disorders and overall life functioning in pediatric populations. At a clinical level, it is often the impact on functioning that motivates people to seek treatment for anxiety, and reduced life impairment is often the primary outcome goal for families (Creswell et al., 2021; Rapee et al., 2023). Given this motivation from end-user stakeholders, it was slightly disappointing to note in our review how few studies address the relationship between impairment and QoL and anxiety disorders, relative to the vast literature exploring presenting symptomatology. The natural implications from this review and our related review on the impact of treatment on life impairment (Dickson et al., 2022) are that mental health researchers need to begin routinely including measures of impairment and QoL into both basic research and clinical trials for pediatric anxiety. Particular advances are likely to come from not only self-reported impairment, but also the inclusion of objective measures (such as school attendance) and independent raters (such as teachers or sports coaches). Even greater value would come from longitudinal studies that evaluate the cascading impact of specific impairments on cognitive and social development. By extending the evidence base on the many and varied ways in which anxiety disorders can impact a child or adolescent’s life, treatment and prevention programs can begin to be more finely tailored.

## Appendix A

See Table

**Table 2:** Search strategies for all databases

**Ovid PsycINFO and Medline**	
(anxiety or anxiety disorder or anxiety diagnosis or generali#ed anxiety or GAD or separation anxiety or social* anxi* or social* fear* or social* phobi* or selective mut* or elective mut* or phobic disorder or phobia or panic disorder or agoraphobi*).ti,ab. AND (Child* or adolescen* or p?ediatric* or teen* or youth or young or school-aged).ti,ab. AND ((life adj2 impairment) or (life adj2 impact) or (life adj1 interfere*) or (daily adj2 functioning) or (global adj1 functioning) or (general adj1 functioning) or (psychosocial* adj2 functioning) or (function* adj1 impair*) or (real#life adj2 outcome) or (real#life adj2 impact) or (adaptive adj2 functioning) or Columbia impairment scale or Children's Global Assessment Scale or Adolescent Life Interference Scale or Child Anxiety Impact Scale or Child Anxiety Life Interference Scale or Sheehan Disability Scale).mp. AND (Quality of life or QoL or HRQOL or (life adj2 satisfaction) or (subjective adj2 well?being) or PedsQL or QoL-ChYMH or PQ-LES-Q).mp	
**PubMed**	
(("anxiety"[Title/Abstract] OR "anxiety disorder"[Title/Abstract] OR "anxiety diagnosis"[Title/Abstract] OR "generalised anxiety"[Title/Abstract] OR "separation anxiety"[Title/Abstract] OR "social anxi*"[Title/Abstract] OR "social fear"[Title/Abstract] OR "social phobi*"[Title/Abstract] OR "selective mutism"[Title/Abstract] OR "elective mutism"[Title/Abstract] OR "phobic disorder"[Title/Abstract] OR "phobia"[Title/Abstract] OR "panic disorder"[Title/Abstract] OR "agoraphobia"[Title/Abstract]) AND ("child*"[Title/Abstract] OR "adolescen*"[Title/Abstract] OR "pediatric"[Title/Abstract] OR "paediatric"[Title/Abstract] OR "teen*"[Title/Abstract] OR "youth"[Title/Abstract] OR "young"[Title/Abstract] OR "school-aged"[Title/Abstract])) AND ("quality of life" OR "qol" OR "hrqol" OR "life satisfaction" OR "subjective well-being" OR "pedsql" OR "qol-chymh" OR "PQ-LES-Q" OR "life Impairment" OR "life impact" OR "life interference*" OR "daily functioning" OR "global functioning" OR "general functioning" OR "functional impairment" OR "real-life outcome" OR "real-life impact" OR "adaptive functioning" OR "Columbia impairment scale" OR "Children's Global Assessment Scale" OR "Child Anxiety Impact Scale" OR "Sheehan Disability Scale")	
**Web of Science**	
anxiety or “anxiety disorder” or “anxiety diagnosis” or “generali?ed anxiety” or “separation anxiety” or “social* anxi*” or “social* fear” or “social* phobi*” or “selective mutism” or “elective mutism” or “phobic disorder” or phobia or “panic disorder” or agoraphobia (Abstract) AND child* or adolescen* or pediatric or paediatric or teen* or youth or young or “school-aged” (Abstract) AND "life Impairment" OR "life impact" OR "life interfere*" OR "daily functioning" OR "global functioning" OR "general functioning" OR "functional impairment" OR "real-life outcome" OR "real-life impact" OR "adaptive functioning" OR "Columbia impairment scale" OR "Children’s Global Assessment Scale" OR "Adolescent Life Interference Scale" OR "Child Anxiety Impact Scale" OR "Child Anxiety Life Interference Scale" OR "Sheehan Disability Scale" or "quality of life" OR qol OR hrqol OR "life satisfaction" OR subjective AND well-being OR pedsql OR qol-chymh OR PQ-LES-Q	

2.

## Appendix B

See Table

**Table 3:** Sensitivity analysis: prioritizing child scale versions over caregiver scale versions

Comparison	Prioritizing Caregiver Scale Versions	Prioritizing Child Scale Versions
Life Impairment	*g* = 3.23, 95% CI = 2.25, 4.21, Z = 6.46, *p* = < .001	*g* = 3.05, 95% CI = 2.06, 4.05, Z = 6.01, *p* = < .001

^3^.

## Footnotes

1 The terms “young people” and “pediatric” will be used to refer to children and adolescents.
